# Screening for language and speech delay in children under five years

**DOI:** 10.1186/s12887-021-02817-7

**Published:** 2021-09-08

**Authors:** Sophie Jullien

**Affiliations:** Barcelona Institute for Global Health, University of Barcelona, Barcelona, Spain

**Keywords:** Screening, Language delay, Speech delay, Preschool child

## Abstract

We looked at existing recommendations and supporting evidence on the effectiveness of universal screening for language and speech delay in children under 5 years of age for short- and long-term outcomes.

We conducted a literature search up to the 20th of November 2019 by using key terms and manual search in selected sources. We summarized the recommendations and the strength of the recommendation when and as reported by the authors. We summarized the main findings of systematic reviews with the certainty of the evidence as reported on the accuracy of the screening tests for detecting language and speech delay, the efficacy of existing interventions for children with language and speech delay, and the potential harms associated with screening and the associated interventions.

Several screening tools are used to assess language and speech delay with a wide variation in their accuracy. Targeted interventions improve some measures of speech and language delay and disorders. However, there is no evidence on the effectiveness of such interventions in children detected by screening with no specific concerns about their speech or language before screening. There is no evidence assessing whether universal screening for language and speech delay in a primary care setting improves short and long-term outcomes (including speech and language outcomes and other outcomes). Finally, there is no evidence on the harms of screening for language and speech delay in primary care settings, and there is limited evidence assessing the potential harms of interventions.

## Background

### Introduction

The World Health Organization (WHO) European Region is developing a new pocket book for primary health care for children and adolescents in Europe. This article is part of a series of reviews, which aim to summarize the existing recommendations and the most recent evidence on preventive interventions applied to children under 5 years of age to inform the WHO editorial group to make recommendations for health promotion in primary health care. In this article, we looked at existing recommendations and supporting evidence on the effectiveness of universal screening for language and speech delay in children under 5 years of age for short- and long-term outcomes.

### Why is the detection of language and speech delay important?

Language is the coding system that permits conceptualisation, reasoning and understanding, while speech is one vehicle for expressing language through combined sounds [1]. Language or speech delay refers to cases where the development of the ability to understand and speak is correct but slower than what is accepted as normal, whereas language or speech disorders refer to cases where the speech or language ability deviate from what is expected as normal development [2, 3]. Language disorders can involve the form (phonology, morphology, syntax), the content (semantics), and the function of language in communication (pragmatics), or in any combination [3]. Speech disorders refer to difficulty with forming specific words or sounds and/or with fluency, needed to communicate with others [2]. Language and speech disorders can exist by themselves or combined [3].

School-aged children with language or speech delay may be at increased risk of learning and literacy disabilities, including difficulties with reading and writing. Children with such conditions may also be at higher risk for behaviour and psychosocial adjustment, which may persist into adulthood [4].

### Context

The median prevalence of isolated speech and language delays and disorders (this is without associated developmental delay, autism spectrum disorder or intellectual disability) was estimated at 6% (range from 5 to 12%) among children between two and 5 years of age in the United States [3, 4].

Language and speech are two of the main domains of child development, or neurodevelopment, together with gross and fine motor skills, social and personal skills, activities of daily living, and cognition. These domains are characterized by continua, this is that one end of the diagnostic spectrum has a border with normality [5]. Language and speech disorders can occur with other developmental disabilities, such as autism spectrum disorder, or with emotional or behavioural disorders, such as attention deficit hyperactivity disorder (ADHD), and might be detected as early manifestations of such disorders [2]. Early identification of children with language and speech delay and disorder would allow interventions at an early stage, before these problems interfere with learning abilities and behavioural adjustment, to reach better health, academic and social outcomes [3]. Universal screening of all preschool children has been suggested to this end, for early detection and intervention and potentially better outcomes [6]. However, the identification of children with language and speech delay through universal screening is challenging. Cultural, socioeconomic and contextual factors make these children a variegated group, which is difficult to evaluate with a simple screening tool [7].

As reminded by the Canadian Task Force on Preventive Health Care, “screening differs from developmental surveillance, which refers to ongoing monitoring by clinicians of a child’s development, identification of risk factors and elicitation of parental concerns” [6].

Finally, although hearing loss is related to language and speech delay, we do not address universal screening for hearing loss in newborns in this document.

## Key questions

1. How accurate are the screening tests for detecting language and speech delay in children under 5 years of age?
2. Are the interventions for children identified with language and speech delay effective for improving (short- and long-term) language and speech outcomes?
3. Does screening programme for detection and early intervention of language and speech delay in children younger than 5 years improve short- and long-term outcomes?
4. What are the potential harms of screening and interventions for language and speech delay for children and their family?

## Search methods and selected manuscripts

We described the search methods, data collection and data synthesis in the second paper of this supplement (Jullien S, Huss G, Weigel R. Supporting recommendations for childhood preventive interventions for primary health care: elaboration of evidence synthesis and lessons learnt. BMC Pediatr. 2021. 10.1186/s12887-021-02638-8).

The search was conducted up to the 20th of November 2019, by manual search and by using the search terms “language” and “speech”. We included any document that addressed at least one of the key questions. We did not find any relevant document from the WHO. We found recommendations and their supporting evidence from the United States Preventive Services Task Force (USPSTF) (2015). The Centers for Disease Control and Prevention (CDC) addresses “Language and speech disorders in children” in their website, mainly addressed to the general public. They promote observation of the children by their parents concerning the developmental milestones and provide recommendations on what should be done for children identified with speech or language concern. However, we did not find any recommendations from the CDC regarding universal screening. The current recommendation from the UK National Screening Committee (UK NSC) is based on an external review published in 2005. According to their website, they are currently reviewing the recommendations on this topic, although it is also stated that the updated review is estimated to be completed by November 2013.

The Royal College of Paediatrics and Child Health (RCPCH) dedicates a whole chapter on child development in their recent book, which includes a section on screening and speech and language disorders. The PrevInfad workgroup (Spanish Association of Primary Care Pediatrics) (2017) and the Canadian Task Force on Preventive Health Care (2016) developed documents with recommendations and supportive evidence on developmental delay with generic measures covering all aspects of development, but do not address language and speech delay as a single domain for universal screening. For the feasibility of this review, we cite these sources as reference for readers, but we did not summarise them.

The search in the Cochrane library by using the search terms ‘language’ OR ‘speech’ in titles returned 11 reviews and one protocol. By screening the titles and abstracts, we included one review (Law 2003) and one protocol (Law 2017). Although published earlier than 2010, we included the Law 2003 review as we judged it was relevant for this summary document. The protocol we identified is for updating the Law 2003 review. We identified one additional systematic review (Kasper 2011) by hand search of the references of the manuscripts identified by the above methods.

All the included manuscripts for revision in this article are displayed in Table 1.

**Table 1:** Included manuscripts for revision

Sources	Final selected manuscripts
**WHO**	None
**USPSTF**	• Siu 2015 – Recommendations [4]
	• Wallace 2015 – Evidence support and systematic review [3], full systematic review [8]
**CDC**	• Language and speech disorders in children (website) [2]
**NICE**	None
**UK NSC**	• UK NSC 2005 – Report and recommendation (currently under review) [9]
**RCPCH**	• Developmental reviews and the identification of impairments/disorders (book chapter with a section on speech and language disorders) [5]
**Cochrane Library**	• Law 2017 - Speech and language therapy interventions for children with primary speech and/or language disorders (Protocol) [10]
	• Law 2003 - Speech and language therapy interventions for children with primary speech and/or language disorders (Review) [11]
**Other systematic reviews**	• Kasper 2011 - Population-Based Screening of Children for Specific Speech and Language Impairment in Germany: A Systematic Review [12]
**Sources for developmental delay**	• PrevInfad 2017 – Early detection of developmental disorders (Recommendations and supporting evidence) [13]
	• Canadian Task Force on Preventive Health Care 2016 - Recommendations on screening for developmental delay [6]

*Abbreviations*: *CDC* Centers for Disease Control and Prevention, *NICE* National Institute for Health and Care Excellence, *RCPCH* Royal College of Paediatrics and Child Health, *UK NSC* UK National Screening Committee, *USPSTF* US Preventive Services Task Force, *WHO* World Health Organization

## Existing recommendations

We summarized the existing recommendations and the strength of recommendations as per their authors in Table 2.

**Table 2:** Summary of existing recommendations

Source	Ref	Date	General recommendations for language and speech delay screening
**USPSTF**	[4]	2015	“The USPSTF concludes that the current evidence is insufficient to assess the balance of benefits and harms of screening for speech and language delay and disorders in children aged 5 years or younger. (I statement)”
**RCPCH**	[5]	2019	“When concerns are raised, appropriate tools should be used to aid assessment. (strong evidence)”
**UK NSC**	[9]	2005	“Systematic population screening programme not recommended” (Currently under revision)
**Canadian Task Force**	[6]	2016	“We recommend against screening for developmental delay using standardized tools in children aged 1 to 4 years with no apparent signs of developmental delay and whose parents and clinicians have no concerns about development (strong recommendation; low- quality evidence)”

*Abbreviations*: *RCPCH* Royal College of Paediatrics and Child Health, *UK NSC* UK National Screening Committee, *USPSTF* US Preventive Services Task Force

## Existing evidence

The USPSTF commissioned a systematic review of the latest evidence on screening for speech and language delays and disorders in children under 5 years of age, to update their 2006 recommendations of screening in a primary care setting [3, 8]. The review focused on screening children under 5 years of age who have not been previously identified with another disorder or disability that may cause speech or language impairment. The review authors assessed screening instruments specific to speech and language conditions, but also more general developmental screening tools with speech and language components. Another inclusion criterion was that screening tools needed to be feasible and interpretable within a primary care setting [4]. The review authors included randomized controlled trials (RCTs), systematic reviews, and cohort studies of screening and surveillance for speech and language delays and disorders, where children who screened positive received formal diagnostic assessment for speech and language delays and disorders by the age of 6 years. The literature search was conducted up to July 2014.

Another systematic review aimed to evaluate the effectiveness of universal screening for specific language impairment in preschool children in German [12]. To this end, and similarly to the methodological approach of the USPSTF review, the question was divided into a review of the evidence from studies evaluating screening programmes, diagnostic tools, and speech and language interventions. The literature search was conducted up to May 2008.

In the RCPCH book, the authors described general points regarding the diagnosis, screening and other considerations on developmental delay. They focused on several domains of child development that they considered were needed to be checked [5]. The first domain they addressed is “Speech and language disorders”.

### Risk factors

Although a focused research question on the identification of potential risk factors for speech and language disorders is beyond the scope of this summary document, we judged it relevant to report those identified by Wallace et al., the review commissioned by the USPSTF [3]. The USPSTF systematic review included 31 cohort studies (24 with multivariate analysis to control for other factors) and one review of studies on characteristics of late-talking toddlers. The review authors identified male gender, family history of speech or language impairment, lower levels of parental education, and various perinatal risk factors (e.g., prematurity, birth difficulties, and low birth weight) as potential risk factors for speech and language disorders.

### Accuracy of the screening tests for detecting language and speech delay in children younger than 5 years

The systematic review conducted by Wallace et al. evaluated four key questions to assess the accuracy of screening tools for the identification of children in the primary care setting for diagnostic evaluations and interventions: (1) “What is the accuracy of these screening techniques and does it vary by age, cultural/linguistic background, whether it is conducted in a child’s native language, or by how the screening was administered (i.e., parent report, parent interview, direct assessment of child by professional)?”; (2) “What are the optimal ages and frequency for screening?”; (3) Is selective screening based on risk factors (i.e. targeted screening), more effective than unselected, general population screening (i.e. universal screening)?; and (4) “Does the accuracy of selective screening vary based on risk factors? Is the accuracy of screening different for children with an inherent language disorder compared with children whose language delay is due to environmental factors?”

The review authors found no studies addressing the key questions 2, 3, and 4. They included 24 studies addressing the first key question, five good- and 19 fair-quality studies. The included studies evaluated the accuracy of 20 different screening tools, seven screening tools administered by parents, and 13 by trained examiners. Studies were conducted in the US (14 studies), the UK (six studies), Australia, Canada, Germany and Sweden. The review authors summarized the characteristics of included studies in supplementary tables and present the accuracy of findings separately for screening tools administered by parents and by trained examiners [3]. The performance characteristics varied widely. Overall, the screening tools administered by parents performed better than those administered by trained examiners. Screening tools for detecting a true speech and language delay or disorder reported by parents presented a median sensitivity of 81% (range from 50 to 94%) and a median specificity of 87% (range from 45 to 96%). Positive predictive values (PPV) ranged from 18 to 92%, and negative predictive values (NPV) ranged from 67 to 98%. When reported by trained examiners (nurses, primary care providers, teachers or paraprofessionals), the screening tools showed a median sensitivity of 74% (range from 17 to 100%) and a median specificity of 91% (range from 46 to 100%). PPV ranged from 6.6 to 100% and NPV ranged from 89 to 100% (except for one study with a reported NPV of 15%).

In conclusion, “the USPSTF found inadequate evidence on the accuracy of screening instruments for speech and language delay for use in primary care settings” [4]. “No one instrument clearly demonstrated the best characteristics or one age as optimal for screening” [3]. In addition, the authors highlighted the difficulties in comparing the performance of screening tools because of the heterogeneity in terms of screening tools used, populations screened and settings [4].

Kasper et al. found no studies that evaluated diagnostic instruments for specific language impairment in the German language [12].

For the RCPCH chapter on “Developmental reviews and the identification of impairments/disorders”, the authors reviewed the literature up to 2019 [5]. It is worth citing a paragraph from this chapter: “To date, no neurodevelopmental assessment beyond the neonatal period has been generally acknowledged to meet the WHO/Wilson and Jungner criteria for screening programmes. Screening approaches have been examined in relation to autism, language disorders, and conduct disorder, but key criteria have not been met: in particular, the requirements for a sensitive and specific screening test, for cost-effectiveness, and for evidence that early intervention produces better outcomes than waiting until problems manifest themselves before intervening. This lack of evidence for early intervention may appear counterintuitive in the context of knowledge that brain plasticity and thus potential gains are greater in younger children. In general, neurodevelopmental screening has failed to meet the WHO screening criteria because of lack of evidence of effectiveness, rather than evidence of lack of effectiveness. While it is possible to evaluate how well a screening test functions in a relatively small constrained population, it is much more difficult to carry out gold standard tests in large populations and it can also be challenging to follow up large groups of children to establish the productivity of a screening procedure over time” [5]. Finally, “while it is tempting to focus on the accuracy of the assessments employed for the identification of difficulties, it is important to stress that the conversations between professional and parent or carer about a child’s development should, if possible, be founded on an existing trusting relationship between the two parties” [5].

### Effectiveness of interventions targeting young children with language and speech delay in short- and long-term outcomes

There is a wide range of interventions for children with speech and language delay and disorders, which include speech-language therapy sessions and assistive technology [4].

Wallace et al. identified 13 RCTs and one systematic review that evaluated the effect of speech and language interventions on speech outcomes. Four RCTs were conducted in the US, three in Australia, three in the UK, two in Canada and one in New Zealand. Two RCTs were judged to be of good quality, and the remaining 11 and the systematic review of fair quality. The review authors summarized the characteristics of the included studies and outcomes in supplementary tables. They found that most of the included trials showed significant positive results of treating young children with language delays and disorders (6 of the 11 trials) or speech sounds problems (6 of the 8 trials) and treating toddlers and pre-school children for fluency problems (2 of the 2 trials) [3]. However, the review authors described multiple factors that limit their confidence in the interpretation of these findings. The evidence comes from small trials, with a lack of replicated positive findings for most treatment approaches and a lack of data regarding compliance to treatment. The review authors could not perform a meta-analysis because there was a high degree of heterogeneity between the trials regarding the age of the children, the interventions (different agents, intensity, content and strategies), the outcome measures, and the way results were reported. In addition, the applicability of this evidence to universal screening in a primary care setting is also limited. Indeed, the identified trials “did not report treatment effectiveness in children whose speech and language delay had actually been detected by screening; instead, the delays had often been identified as a result of parent or teacher concerns”, and most studies were conducted in populations with a high prevalence of speech and language disorders [3]. The USPSTF also looked at the effect of speech and language interventions on other outcomes. They identified five studies with inconsistent findings on outcomes including socialization, reading comprehension, parental stress, and child well-being or attention level [3]. In conclusion, the USPSTF authors found evidence that interventions improve some measures of speech and language for some children. However, they found inadequate evidence on the effectiveness of such interventions for speech and language delay and disorders among children detected by universal screening, and on their effectiveness on outcomes not specific to speech (e.g., academic achievement, behavioural competence, socioemotional development, and quality of life) [4].

An older Cochrane review was conducted to examine the effectiveness of speech and language interventions for children with primary speech and language delay and disorder [11]. This review is currently being updated [10]. In the review published in 2003, authors included RCTs evaluating children or adolescents with primary speech and language delay/disorder who received “any type of intervention designed to improve an area of speech or language functioning concerning either expressive or receptive phonology (production or understanding of speech sounds), expressive or receptive vocabulary (production or understanding of words), or expressive or receptive syntax (production or understanding of sentences and grammar)” [11]. They identified 36 papers, of which 25 contributed to the meta-analysis. Eight of these papers were also included in the systematic review conducted by Wallace et al. (the remaining seven trials included in the Wallace review were published after the 2003 Cochrane review). Law et al. found that speech and language interventions are effective for children with phonological or vocabulary difficulties but that there is less evidence concerning the effectiveness of these interventions for children with receptive difficulties, and mixed findings concerning the effectiveness of expressive syntax interventions. There were no significant differences between intervention administered by trained parents and professionals. Like the review conducted by Wallace et al., they found a high degree of heterogeneity between included studies, and applicability of the findings to children with speech and language delay detected by universal screening is limited (all included studies were conducted in children already diagnosed with a speech and language delay or disorder).

The German review identified 16 RCTs, including seven trials already included in both reviews by Wallace et al. and by Law et al., and five trials included in one of the two reviews [12]. Overall, the review authors found positive effects from language therapies in the short term, but no evidence of benefits from earlier treatment initiation.

The RCPCH did not identify more recent evidence to add on the findings from the USPSTF review, but “Gillberg makes the valuable point that it is not good enough to ‘wait and see’ how developmental problems will unfold: around two-thirds of children with significant language delay at 30 months will manifest a range of significant associated neuropsychiatric problems as they grow older and many of these problems are likely to benefit from early intervention” [5].

### Benefits of universal screening programmes and early intervention

Kasper et al. identified one study (reported in two manuscripts) evaluating speech and language screening, although authors “did not explicitly report results for children with specific language impairment” and therefore “it is not clear to what extent the study results apply to the children in the focus of this review” [12]. Overall, the review authors concluded that there was no evidence of benefits of universal screening of preschool children with specific language impairment [12].

Wallace et al. identified no study that determined whether universal screening for language and speech delay improves language and speech or other outcomes [3]. There is a “critical need for studies specifically designed and executed to address whether universal screening for speech and language delay and disorders in young children in primary care settings leads to improved speech, language, or other outcomes” [4].

On this aspect, authors from the RCPCH say: “One area where screening is recommended by some authors is universal screening for speech and language followed by appropriate targeted intervention. The problem is that there is still insufficient evidence to support the recommendation of screening. There are a number of reasons for this including the variability of the gold standard measures against which screening tests are evaluated, the tendency for such measures to both under-refer (low sensitivity) and over-refer (low specificity), and the difficulty of establishing predictive validity when the trajectory of language development can be so variable especially in early years – exactly when such measures are commonly recommended” [5].

### Potential harms of screening and interventions for language and speech delay for children and their family

The potential harms of screening and interventions for speech and language disorders in young children in primary care “include the time, effort, and anxiety associated with further testing after a positive screen, as well as the potential detriments associated with diagnostic labelling” [4].

We found no studies that assessed the potential harms of screening for language and speech delay and disorders. Wallace et al. identified three studies that examined adverse effects of interventions and reported no negative impacts on children or parents [3]. None of the studies included in the systematic review conducted by Kasper addressed side effects or undesired effects of speech and language interventions [12]. The USPSTF found inadequate evidence on the harms of universal screening and interventions for speech and language delay and disorders in children aged 5 years or younger [4].

## Summary of findings

- Several screening tools are used to assess language and speech delay in primary care settings, with a wide variation in their accuracy. The USPSTF found no single screening tool with the best characteristics for screening.
- There is evidence that targeted interventions improve some measures of speech and language delay and disorders. However, there is no evidence on the effectiveness of such interventions in children detected by universal screening, this is screening all children with no specific concerns about their speech or language before screening.
- There is no evidence on whether universal screening programmes for detecting language and speech delay for early treatment improves short and long-term outcomes (including speech and language outcomes and other outcomes).
- Potential harms of screening for language and speech delay include burden for the families in terms of time and resources. However, there is no evidence on the harms of universal screening for language and speech delay, and there is limited evidence assessing the potential harms of interventions.
- Well-designed trials evaluating the most accurate screening tool and looking at benefits of universal screening for language and speech delay in young children are needed.

## Data Availability

Not applicable.

## Abbreviation

.: See Jullien S, Huss G, Weigel R. Supporting recommendations for childhood preventive interventions for primary health care: elaboration of evidence synthesis and lessons learnt. BMC Pediatr. 2021. 10.1186/s12887-021-02638-8.
